# Increase of tensile strength and toughness of bio-based diglycidyl ether of bisphenol A with chitin nanowhiskers

**DOI:** 10.1371/journal.pone.0177673

**Published:** 2017-06-12

**Authors:** Mian Wang, Han Xue, Zhiwei Feng, Binfeng Cheng, Haijie Yang

**Affiliations:** School of Life Science and Technology, Henan Collaborative Innovation Center of Molecular Diagnosis and Laboratory Medicine, Xinxiang Medical University, Xinxiang, China; Michigan Technological University, UNITED STATES

## Abstract

It is challenging to reinforce and toughen thermoset epoxy resins. We describe a slurry-compounding technique to transfer a uniform dispersion of chitin nanowhiskers (CW) in ethanol into an epoxy matrix. The incorporation of the hydrophilic CW reinforces the oil-soluble diglycidyl ether of bisphenol A (DGEBA). The resultant CW/epoxy bionanocomposites were transparent and showed considerably enhanced thermal and mechanical properties with tensile strength, modulus, toughness, and elongation at break being increased by 49%, 16%, 457%, and 250%, with only 2.5 wt.% CW. This improvement in strength and toughness is rare for thermoset epoxy/rigid nanofiller systems. We hypothesize that CW with many free amine groups could function not only as a nanofiller but also as a macromolecular polyamine hardener that participates in epoxy curing. The strong covalent interaction between the filler and the matrix allowed for efficient load transfer across the interfaces, which accounted for the greater strength and toughness.

## 1. Introduction

Because of increased attention to ecological safety and green/renewable materials, natural fibers as bio-fillers/reinforcement materials for composites are utilized widely in industry and academia [1–3]. Chitin has desirable mechanical properties, with crystalline regions of α-chitin having a Young’s modulus of as high as 41 GPa based on X-ray diffraction measurements [4]. Because CW is soluble in dilute aqueous acetic acid solution, it has been used to reinforce various water-soluble polymeric materials by solvent casting or impregnation. For example, the Young’s modulus of polyvinyl alcohol fibers has been increased from 28 GPa to 50 GPa by incorporating 30% CW [5]. Chitosan nanocomposites could also be strengthened by incorporating α-CW [6,7]. However, for oil-soluble organic polymers, it is a challenge to prepare homogeneous polymer/CW or CS systems due to inherent immiscibility between the filler and the matrix. In literature, there are several studies on CS strengthened natural rubber, epoxy-functionalized PE, poly(ethylene glycol) diglycidyl ether, and epoxy-terminated polydimethylsiloxane, LDPE etc. [8–15]. In most cases, the addition of CS usually improves only the strength and modulus, with toughness or the elongation at break being reduced remarkably.

Prior to the addition of CW into polymer matrices, raw CW need to be separated into uniform CW bundles with diameters as small as possible [2]. Effective methods have been developed to disperse CW/CS in water/organic solvents uniformly, including acid hydrolysis, 2,2,6,6-tetramethylpiperidine-1-oxyl radical (TEMPO) mediated oxidation, ultrasonication, electrospinning, ionic liquids, and mechanical treatment to produce CW/CS with varying degrees of deacetylation [3]. During the dispersion process, both the chemical and mechanical methods could destroy the crystalline region of CW to varying extents, which is as important as its amorphous structure. The crystalline CW is critical to exploiting its high modulus and strength, and the amorphous structure could be modified to improve the miscibility between the filler and the matrix [4].

Toughness is the ability of any material to deform plastically and to absorb energy during fracturing [16]. To toughen brittle thermoset epoxy, different filler particles such as rubber, core-shell, hyper-branched polymer, thermoplastic and inorganic particles were utilized and various degrees of success were obtained [17–20]. CW is hydrophilic and can be dispersed uniformly in DI water to form a stable suspension. However, transferring this CW dispersion state into oil-soluble diglycidyl ether of bisphenol A (DGEBA) is challenging via either solvent casting or impregnation. To overcome this obstacle, a “slurry-compounding” technique [21, 22] was modified to produce transparent epoxy/CW bionanocomposites (with CW content up to 2.5 wt.%). To the best of our knowledge, a bio-based thermoset diglycidyl ether of bisphenol A (DGEBA)/CW nanocomposite with simultaneously improved strength and toughness has not yet been reported.

## 2. Experimental

### 2.1. Materials

The polymer matrix used is epoxy DER 332 (Dow Plastics), diglycidyl ether of bisphenol A (DGEBA), with an epoxide equivalent weight of 171–175 g/equiv, and density of 1.16g/mL. Chitin from shrimp shell in powder was obtained from Sigma-Aldrich in practical grade. The di-amine curing agent is Ethacure LC-100 from Albemarle Corp., which is a mixture of two diethyltoluenediamine (DETDA) isomers (75–81% 2,4-isomer and 18–20% 2,6-isomer). All other chemical agents and solvents were purchased from Sigma-Aldrich and used as received.

### 2.2. Sample preparation

#### Synthesis of chitin nanowhisker

CW was synthesized following the procedures reported previously with some modification [3]. The raw chitin power was treated with 1M NaOH at room temperature for 1 day, then with 0.3% NaClO2 at 80°C for 3h, and washed with copious water after each step. The purified chitin was suspended in 3N HCl at 100°C for 90 min under stirring, followed by dilution with DI water and centrifugation. This process was repeated three times, and the chitin suspension was dialyzed against DI water overnight for three times and centrifuged. The final chitin nanowhisker slurry was re-dispersed in ethanol at concentration of 1g/mL and stored at 4°C for further use.

#### Preparation of CW/epoxy bionanocomposites

CW/epoxy bionanocomposites were prepared via slurry compounding. For example, to prepare epoxy nanocomposite with 1 wt% CW, 1g of CW in 1ml ethanol suspension was added into 78.4 g of DGEBA in a beaker and the mixture was heated at 60°C under stirring until no bubble came out. During the mixing process, the CW/DGEBA mixture turned from a little turbid to white transparent, indicating CW being well mixed with epoxy. Then 20.6 g amine hardener 100-LC (DEGBA: LC-100 = 3.8:1, w/w, this ratio is set for all CW/epoxy nanocomposites) was added into the CW/DGEBA and homogenized at 2000 rpm for 30 min. Last, the three component CW/DGEBA/LC-100 mixture was degassed under vacuum at 80°C for 1h and cured at 100°C for 2h and at 200°C for 5h. The curing condition was carefully determined with the help of rheometer to make sure the completion of the process. This slurry compounding method is efficient and cost-effective in terms of time and amounts of solvents required for the preparation and thus eco-friendly and economically. Three CW/epoxy nanocomposites were prepared and denoted as CW/Epoxy1, CW/Epoxy2 and CW/Epoxy3 at CW loadings of 0.5, 1, 2.5 wt%.

### 2.3. FTIR and ATR-FTIR

FTIR spectra were acquired with a Perkin-Elmer 2000 spectrometer operated in the absorbance mode over a scan range of 4000 to 400 cm^-1^. Before FTIR testing, CW was washed with ethanol and water three times each, then dried at 60°C for 24 h. CW/epoxy nanocomposites were evaluated in ATR-FTIR mode.

### 2.4. X-ray diffraction

XRD patterns were obtained using a Bruker X-ray diffractometer (equipped with a two-dimensional detector) in reflection mode. Test was carried out using nickel filtered Cu Kα radiation (λ = 0.15418 nm) under a voltage of 40 kV and a current of 40 mA.

### 2.5. FE-SEM

The fracture surface of the CW/epoxy bionanocomposite samples after tensile test was observed with a field emission-scanning electron microscopy (Agilent 8500 FE-SEM) without metal sputter coating.

### 2.6. Transmission electron microscopy

High resolution TEM micrograph was taken with a Philips CM300 at 300 kV. A drop of CW/Ethanol solution was deposited on a 200 mesh copper grid and vacuum dried before observation.

### 2.7. Thermogravimetric analysis

Thermal stability of CW/epoxy nanocomposites was evaluated with a thermo-gravimetric analyzer (TA instrument Q500) at a heating rate of 10°C/min from room temperature to 800°C under air. The degradation temperatures of the nanocomposites were determined as the minimum point of the derivative weight versus temperature.

### 2.8. Dynamic mechanical analysis

Dynamic mechanical properties were examined with a TA Instruments DMA 2980 operated in the single cantilever bending mode at an oscillation frequency of 1.0 Hz and a scanning rate of 3°C/min. The samples for DMA tests were cut into rectangular bars having dimensions (L×W×T) of 35 mm × 5 mm × 3 mm. A minimum of 3 specimens of each composition were tested. The glass transition temperature (*T*_g_) was assigned as the maximum of the tan δ.

### 2.9. Tensile test

Tensile properties were determined using the Instron 5569 testing machine according to the ASTM standard D638-03 at a speed of 1mm/min at room temperature. The specimens were cut into dog-bone shape with dimensions (L×W×T) of 55 mm× 10 mm × 3 mm. A minimum of 8 specimens of each composition were tested, and the mean vale and standard deviation were calculated.

### 2.10. Statistical analysis

Data were expressed as mean ± standard deviation (SD). Student t test or two-way analysis of variance (ANOVA) was employed to analyze the difference between groups. A p-value < 0.05 was considered statistically significant. *p < 0.05 versus control group.

## 3. Results and discussion

### 3.1. Fabrication of bionanocomposites based on epoxy DGEBA and CW via slurry-compounding

Inspired by a study on clay/epoxy nanocomposites [22], a slurry-compounding technique was modified to fabricate CW/epoxy bionanocomposites. When the CW content increased, the precipitation of CW throughout the epoxy matrix was visible during curing at high temperatures. This led to worse thermal and mechanical properties of the final products. Thus, we prepared three samples of bionanocomposites with CW contents no more than 2.5 wt%.

**Fig 1:**
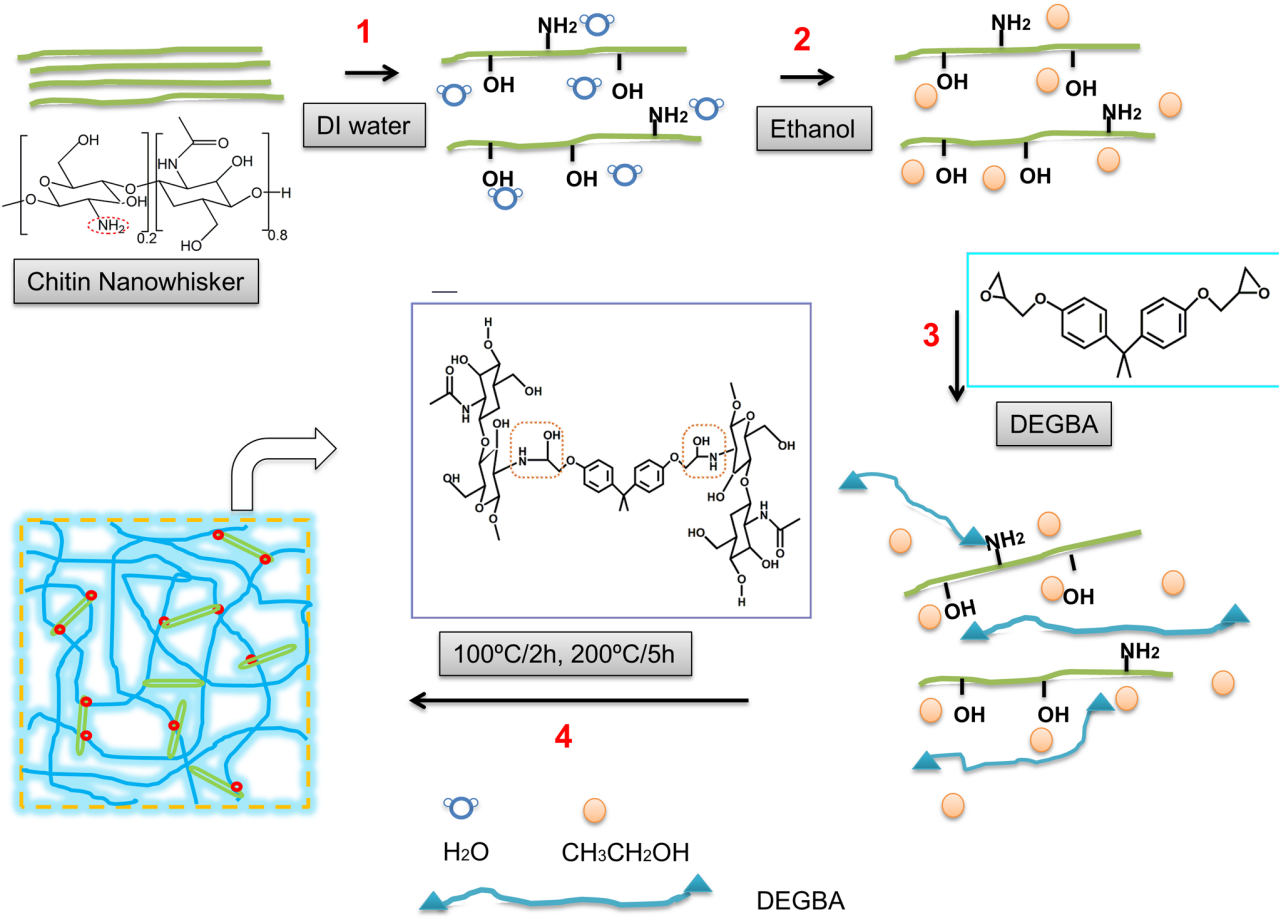
Schematic representation of CW/epoxy bionanocomposites prepared by slurry compounding.

A schematic illustration of the slurry-compounding process is shown in Fig 1. Here, both the choice of solvent and the CW content play important roles in determining the morphology and properties of the CW/epoxy bionanocomposites. For comparison purpose, acetone was also tried as a dispersion media for CW. However, even with addition of 0.5 mL of CW/acetone (1mg/mL), DEGBA (78.7g) quickly turned from transparent to opaque in less than five minutes, with CW precipitating out of DGEBA in step 3. In contrast, DGEBA/CW/ethanol with CW content less than 2.5 wt% remained clear for hours. However, when the CW weight percent exceeds this value, CW began to precipitate in step 4 at 100°C. The reason might be the increased mobility of CW in less viscous DEGBA/CW mixture at higher temperatures, which prompts the probability of collision and aggregation of nanosized CW with higher contents. And the precipitated CW proceeded to evolve into larger aggregates at 200°C in step 4, as the epoxy viscosity increased with time during cure process.

Clearly, the dispersion and maximum loading of CW depends on several parameters, such as the compatibility of the CW and the epoxy, the solvent polarity, and the intensity of mixing, the mixing entropy of all the curing components and the kinetics of the cross-linking reaction, which need to be investigated with more sophisticated methods and instruments. In this study, the slurry-compounding technique uses only water and ethanol to fabricate oil-soluble DEGBA bionanocomposites based on CW. This is a green approach and one of the novelties of this study in terms of cost and the properties of the final product.

### 3.2. Structure and chemical properties of CW and the bionanocomposites

**Fig 2:**
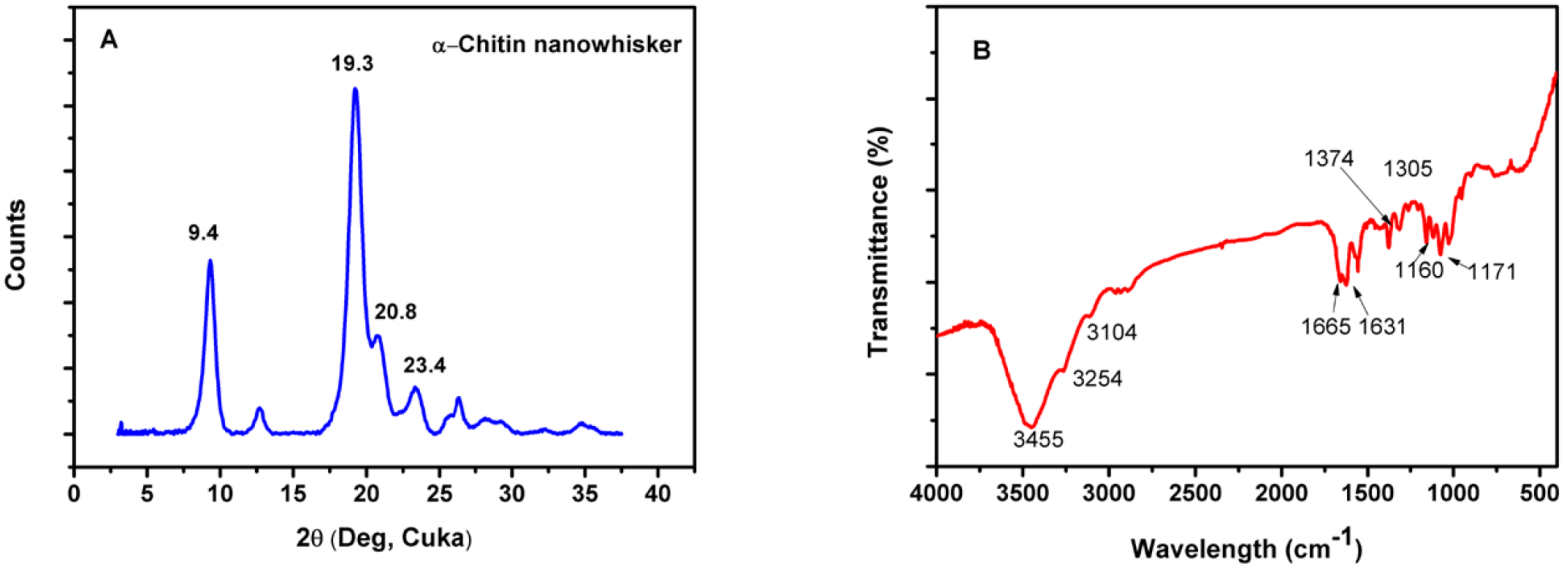
XRD (A) and FTIR (B) spectrum of α-chitin nanowhiker (CW).

Acid hydrolysis of raw chitin produces α-chitin nanocrystals or nanowhiskers. In Fig 2A, the XRD pattern shows four diffraction peaks near 9.4°, 19.3°, 20.8°, and 23.4°, which can be assigned to 202, 110, 120, and 130 planes, respectively [23,24]. These typically correspond to the crystalline structure of α-chitin. The crystallinity index (CrI) was determined according to the method proposed for cellulose and chitosan[25, 26]:

$$\text{rI}=\frac{\text{I }110-\text{Iam}}{\text{I}110}\times 100$$ (1)

where I_110_ is the maximum intensity of the (110) lattice diffraction and I_am_ is the intensity of amorphous diffraction in the same units at 2θ = 16. Thus, the crystalline degree of α-CW prepared in this study is ca. 91%.

In Fig 2B, the FT-IR spectrum of the CW mainly shows characteristic absorption peaks at 3455, 3254, and 3104 cm^-1^, belonging to OH and NH of primary amine, respectively. The absorption peak at 1665 cm^-1^ is commonly assigned to the stretching of the C = O group hydrogen bonded to N-H of the adjacent intra-sheet chain. The absorption peak at 1631 cm^-1^ is attributed to a specific hydrogen bond of C = O in the hydroxyl methyl group of the next chitin residue [27]. The absorption peak at 1558 cm^-1^ originates from the amide II band for chitin [28]. A comparison of the FTIR data between the prepared CW and the raw chitin (data not shown) implies their similarity in chemical structure. The degree of f(%*DA*) was calculated from:

$$\%DA=\frac{\text{A}1655}{\text{A}3455}\times 115\%$$ (2)

where A_1655_ and A_3455_ are the absorbance ratios at 1665 cm^-1^ and 3455 cm^-1^, respectively, proposed by Baxter et al. [29], and was found to be ~20%. Based on this low deacetylation degree and the high CW crystallinity, we hypothesize that most of the bulk crystalline region of CW remained intact, and hydrolysis might occurred on the CW surface.

**Fig 3:**
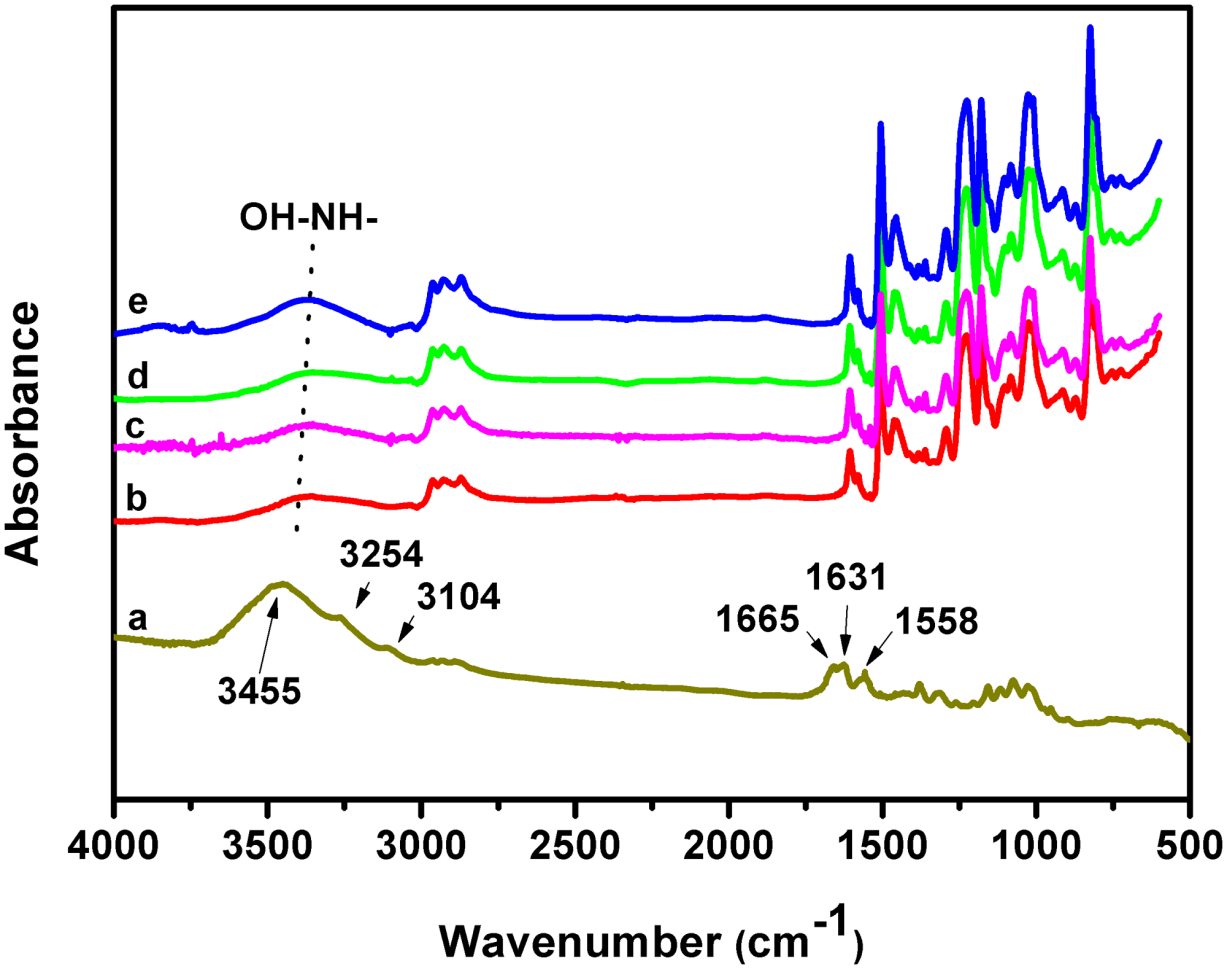
FTIR spectrum of CW (a) and ATR-FTIR spectra of the neat epoxy and CW/epoxy bionanocomposites. (b) Neat epoxy; (c) CW/epoxy1; (d) CW/epoxy2; (e) CW/epoxy3.

Fig 3b–3e shows ATR-FTIR spectra of CW/epoxy bionanocomposites. In the range of 2000–500 cm^-1^, epoxy rings usually show absorptions at 904, 833, and 744 cm^-1^[21], the intensity of which decrease with time in the curing process. However, benzene rings of DGEBA epoxy exhibit characteristic absorption peaks at 1600, 1580, 1500, 1460, 908, 821, 760 cm^-1^[21], as shown in Fig 3 for all epoxy composites. Thus it might be concluded that in the case of DEGBA, the three characteristic bands of epoxy rings cannot be used to monitor curing process. In Fig 1a, a pair of absorption peaks at 3254 and 3104 cm^-1^ belonging to bonded primary amine of CW [30] disappear for CW/epoxy nanocomposites, implying the amine probably participating in the epoxy ring-opening reaction. Additionally, the peak at 1030 cm^-1^ [30], which could be ascribed to C-O or C-N stretching vibrations, is stronger in intensity for CW/epoxy2 and 3 than for neat epoxy and CW/epoxy1, further implying the possible formation of C-N bond between CW and the epoxy matrix. Therefore, we hypothesize that in curing stage at temperatures as high as 200 and 500°C, CW bearing free primary amine might act as a macromolecular hardener for epoxy cure, together with amine hardener LC100. This hypothesis was confirmed in a previous study, in which CS with primary amine was applied as a hardener for epoxy curing [15]. Other than this, CW might also interact with the epoxy matrix via hydrogen bonding, as indicated by the down-shift of the OH absorbance peak in the range of 3600–3300 cm^-1^ and its increased intensity with the addition of CW [31]. Then it could be expected that the synergistic effect of these interactions between CW and the epoxy could enhance load transfer efficiency from the matrix to the filler, leading to improved mechanical properties.

### 3.3. Thermal-mechanical properties of cured epoxy resin and bionanocomposites

**Fig 4:**
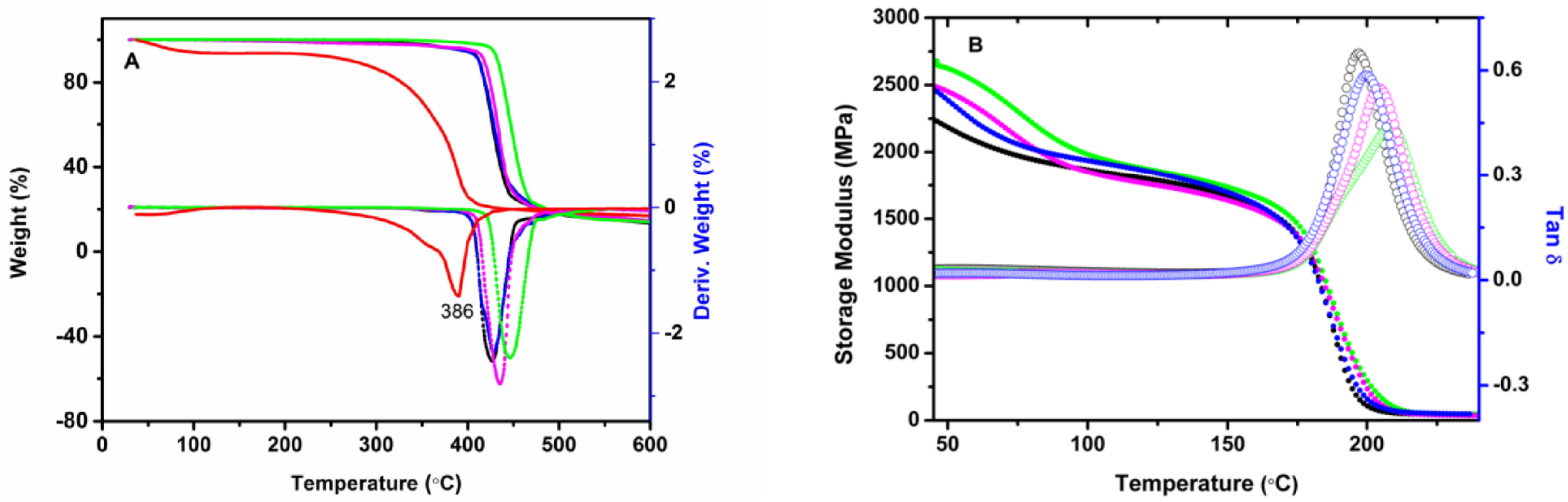
(A) TGA (solid lines) and derivative TGA (dashed lines) curves; (B) DMA (solid circles) and tan δ curves (open circles) of neat epoxy (black) and bionanocomposites: CW (red), CW/epoxy1 (blue), CW/epoxy2 (purple), and CW/epoxy3 (green).

Fig 4A shows that the TGA and derivative TGA curves of CW, neat epoxy and the CW/epoxy bionanocomposites. CW shows a primary backbone decomposition point at 386°C, whereas all epoxy nanocomposites demonstrate one main mass loss at 426–446°C, which corresponds to the degradation of the epoxy network [21]. Specifically, the decomposition point of the neat epoxy increases with CW content, which is elevated by up to 20°C with only 2.5 wt.% CW. In contrast, the addition of CW reduced the degradation temperatures of thermoplastics [32], due to large chitin crystallite surface and weak interactions of α-chitin with the matrix as suggested by the researchers. In our case, we attribute the improved thermal stability of CW/epoxy to the strong interaction between the filler and the matrix.

Fig 4B shows the temperature dependency of the storage modulus (*E*’) and tan *δ* for the neat epoxy resin and CW bionanocomposites. At room temperature, the *E*’ of the epoxy increases with CW content, indicating more rigid nanocomposites formed upon the incorporation of CW. The peak position of tan *δ* corresponds to the glass transition temperature (*T*_g_). Clearly, the *T*_g_ of neat epoxy resin increases with increasing CW content, due to both the steric hindrance of the filler to the epoxy molecules and the interactions between them. The symmetric shape of tan *δ* is indicative of complete curing of neat epoxy and all CW/epoxy nanocomposites. In the temperature range of 100–170°C, a rubbery plateau region of *E’* exists for all samples, implying a tough network structure in all epoxy nanocomposites. On the other hand, the heterogeneity and cross-linking density of the network could be qualitatively assessed with a peak factor, which is defined as the full width at half maximum of the tan *δ* peak divided by its height [33]. Obviously, the peak factor increases with CW content, implying the neat epoxy consisting of a more homogenous and higher cross-linked network structure compared with CW/epoxy nanocomposites. This is understandable, considering more complicated components in the latter.

**Fig 5:**
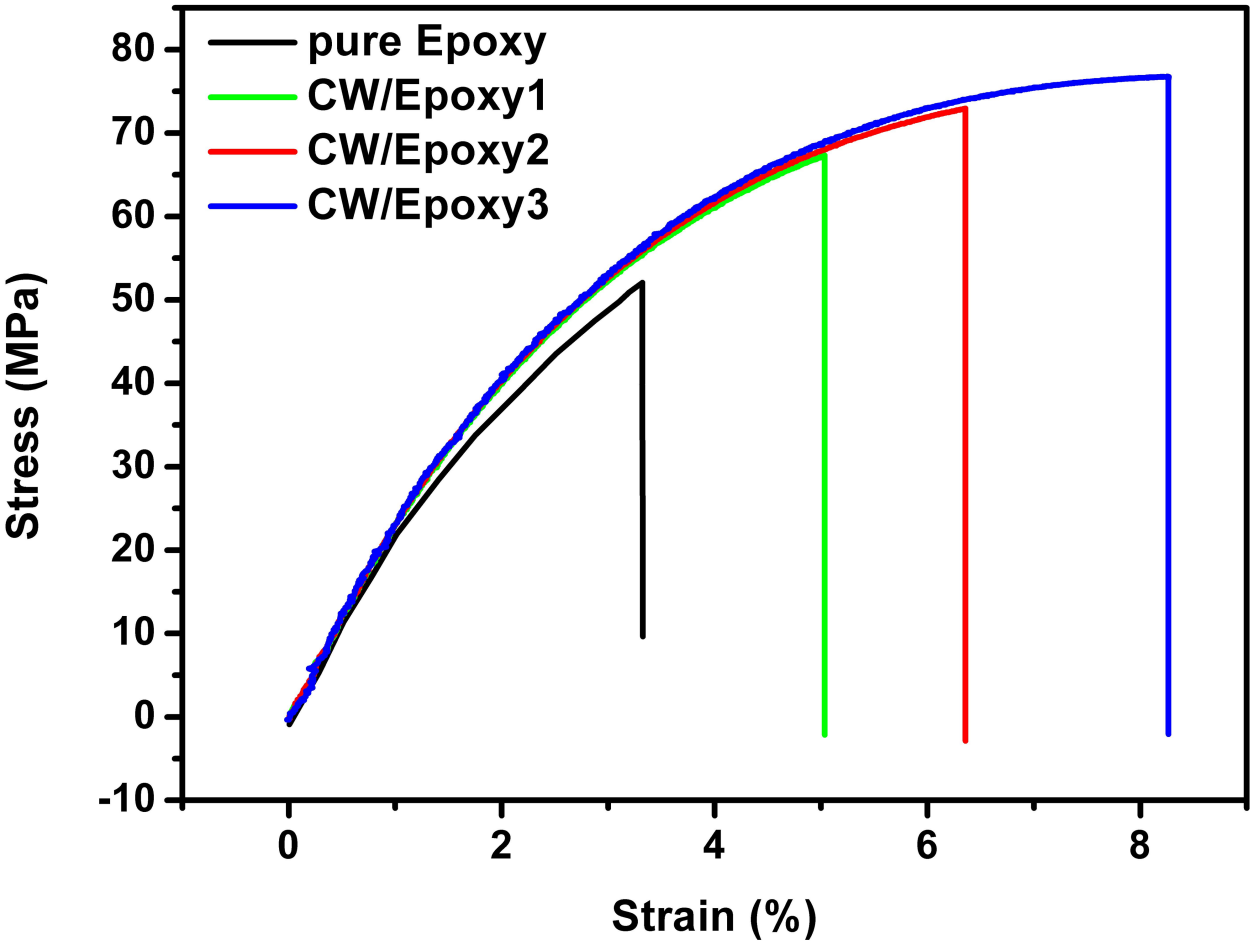
Representative stress-strain curves of neat epoxy and CW/epoxy bionanocomposites.

In this study, acid-hydrolyzed chitin with an appropriate number of amine groups in epoxy functions not only as a strengthening filler but also as a polyamine hardener, as evidenced by FITR measurements. Table 1 summarizes the mechanical data obtained by tensile tests, including Young’s modulus (*E*), the yield strength (σ_y_), the tensile strength at maximum elongation (σ_max_), the strain at break (ε_b_), and the toughness; representative tensile testing curves are shown in Fig 5. All parameters of mechanical properties increase monotonously with CW content. Most remarkably, the addition of only 2.5 wt.% CW enhanced the modulus, strength, toughness, and elongation at break of neat epoxy by 49%, 16%, 457%, and 250%, respectively, which implies that the CW/epoxy bionanocomposites are stronger and tougher than their counterpart.

**Table 1:** Thermal and mechanical properties of cured epoxy and the bionanocomposites.

Sample cod	CW content (%)	Tensile strength (MPa)	Tensile modulus (Mpa)	Elongation at break (%)	Toughness (J.m^-3^10^4^)	Decompositon temperature (Td_max_ °C)	Tg (°C)
Control	0	51.6	2410	3.31	99.7	426.2	197.1
CW/epoxy1	0.5	67.4*	2733*	5.02**	213	430.1	199.9
CW/epoxy2	1.0	72.8**	2760*	6.35**	309	433.5	204.6
CW/epoxy3	2.5	77.1**	2812**	8.27***	456	446.3	208.7

*p < 0.05,

**p < 0.01 and

***p < 0.001 versus control group; control: neat epoxy.

Most studies on CW/polymer nanocomposite systems have shown that the addition of the high crystalline CW led to stiffer materials [32, 34], but reduced elongation at break because of CW restricting the mobility the polymer chains. Even through these CW/polymer nanocomposites became stronger, the polymer matrices broke earlier before reaching their highest yield strengths. This resulted in a lowered capacity of plastic deformation and energy absorbance and therefore decreased toughness. In our study, the CW/epoxy bionanocomposites resist this premature break/fracture via a covalent network structure among CW, epoxy and the hardener. This toughening mechanism is somewhat similar to that for covalent IPN structure formed between PU prepolymer and epoxy.

### 3.4. Morphology of cured epoxy and bionanocomposites

**Fig 6:**
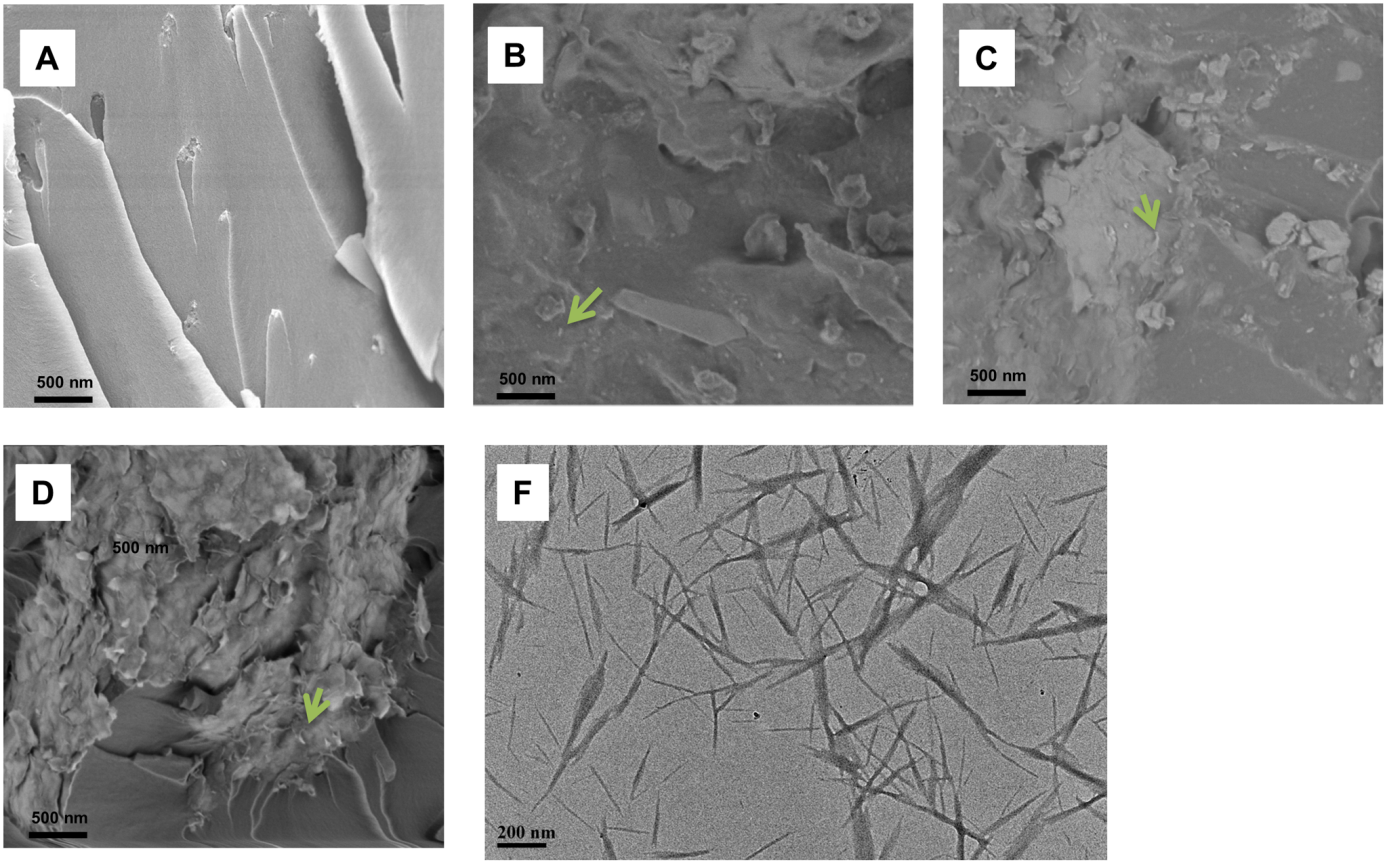
FE-SEM micrographs of the fracture surfaces 292 of neat epoxy (A), CW/epoxy1 (B), CW/epoxy2 (C), CW/epoxy3 (D), and TEM micrograph of CW (E). Green arrows point to the bright dots of the fractured CW embedded in the epoxy matrix.

Fig 6 shows the FE-SEM images of the fracture surface of neat epoxy and its bionanocomposites after tensile tests. The fractured surface of neat epoxy is relatively smooth, indicating the brittleness of the thermoset epoxy. In contrast, the surfaces of the bionanocomposites are much rougher, implying the improved toughness of the epoxy matrix. The observed bright dots in Fig 6B–6D could be the fractured CW embedded in the epoxy matrix, because for the FE-SEM instrument employed in this study, no conductive metal coating was needed, which excluded the possibility of the metal deposition on the epoxy fracture surface as seen with other SEM measurements. Obviously, even with higher CW contents in CW/epoxy2 and 3, there is no prominent CW aggregation. More importantly, nearly no CW is pulled out of the matrix upon stretching, which implies strong interfacial interactions between CW and the epoxy matrix. The size of the CW fibers in ethanol suspension estimated from the TEM image (Fig 6E) agrees well with the CW in the epoxy matrix. This confirms the effectiveness of slurry-compounding in transferring CW dispersion in solution into the epoxy matrix.

## 4. Conclusions

Simultaneously reinforcing and toughening thermoset epoxy resins are challenging due to the brittleness of the epoxy resins. CW-reinforced thermoset epoxy was prepared by a modified slurry-compounding method, which used low amounts of solvents and demonstrated the feasibility and cost-saving nature of this method. The epoxy/CW nanocomposites showed superior thermal and mechanical properties compared with neat epoxy. The mechanism underlying this substantial enhancement in thermo- and mechanical properties is that CWs with free amine functionality could participate in epoxy curing as a macromolecular hardener. The resulting covalent network structure formed between CW and epoxy could enhance load transfer across the matrix/filler interface. This strategy for the choice of the filler and its modification may inspire material scientists to design versatile hardeners to prepare epoxy composites with desired properties.
